# Targeting Insulin-Like Growth Factor-I and Extracellular Matrix Interactions in Melanoma Progression

**DOI:** 10.1038/s41598-017-19073-4

**Published:** 2018-01-12

**Authors:** Berline Murekatete, Ali Shokoohmand, Jacqui McGovern, Lipsa Mohanty, Christoph Meinert, Brett G. Hollier, Alfred Zippelius, Zee Upton, Abhishek S. Kashyap

**Affiliations:** 1 Institute of Health and Biomedical Innovation, School of Biomedical Science, Queensland University of Technology, Brisbane, QLD Australia; 2 Australian Prostate Cancer Research Centre - Queensland, Institute of Health and Biomedical Innovation, Queensland University of Technology, Translational Research Institute, Brisbane, Queensland Australia; 3 Institute of Medical Biology, Agency for Science, Technology and Research (A*STAR), Singapore, Singapore; 4 Cancer Immunology, Department of Biomedicine, University Hospital Basel and University of Basel, Basel, Switzerland

## Abstract

Insulin-like growth factor (IGF)-I binds to the ECM protein vitronectin (VN) through IGF binding proteins (IGFBPs) to enhance proliferation and migration of skin keratinocytes and fibroblasts. Although evidence exists for the role of individual components of the complex (IGF-I, IGFBP-3 and VN), the cellular functions stimulated by these proteins together as a complex remains un-investigated in melanoma cells. We report here that the IGF-I:IGFBP-3:VN trimeric complex stimulates a dose-dependent increase in the proliferation and migration of WM35 and Sk-MEL28 melanoma cells. In 3D Matrigel^™^ and hydrogel cultures, both cell lines formed primary tumor-like spheroids, which increased in size in a dose-dependent manner in response to the trimeric complex. Furthermore, we reveal IGFBP-3:VN protein complexes in malignant melanoma and squamous cell carcinoma patient tissues, where the IGFBP-3:VN complex was seen to be predominantly tumor cell-associated. Peptide antagonists designed to target the binding of IGF-I:IGFBP-3 to VN were demonstrated to inhibit IGF-I:IGFBP-3:VN-stimulated cell migration, invasion and 3D tumor cell growth of melanoma cells. Overall, this study provides new data on IGF:ECM interactions in skin malignancies and demonstrates the potential usefulness of a growth factor:ECM-disrupting strategy for abrogating tumor progression.

## Introduction

The high mortality rate of melanoma is associated with the metastasis of malignant melanoma cells to critical organs of the body^1^. Insulin-like growth factor-I (IGF-I), amongst others, is known to enhance tumor growth and invasion^2^. IGF-I can act as a paracrine factor that drives malignant cell transformation through the activation of the IGF type-I receptor (IGF-IR)^3^. All melanocytic cells express the IGF-IR, with increased expression correlated with disease progression^4,5^. In addition, growth factor interactions with the extracellular matrix (ECM) play important roles in tumor biology, facilitating tumor cell attachment, proliferation and invasion^6,7^, and resistance against chemotherapeutic drugs^8^.

Proteins in the IGF system have been shown to interact with ECM proteins such as fibronectin (FN), vitronectin (VN), laminins, as well as integrins, which in turn, modulate the function of IGF-I^9,10^. Previous studies have demonstrated that IGF-I interacts with VN through IGFBPs to form IGF-I:IGFBP:VN trimeric (TRI) complexes^11^. Further, IGFBP:VN complexes have been observed in tumor biopsies from breast cancer patients, associating with the invasive front of tumor clusters and around tumor blood vessels^12^. This is aligned with the concept that VN is a matricellular protein that functions as a scaffold onto which growth factors, such as IGF-I, are captured, exposing cells to concentrated foci of growth factors available for receptor stimulation^13^. Indeed, complexes of TRI have been shown to promote enhanced cell attachment and migration, as well as protein synthesis, in human keratinocytes^14^ and breast cancer cell lines^11,15,16^.

In melanoma VN is known to stimulate cell migration and invasion^17,18^ through the α_v_β_3_ integrin, the key VN-binding receptor frequently over-expressed in both vertical growth phase (VGP) and malignant melanoma tumors^19^. However, to our knowledge no published studies address the impact of growth factor interactions with the ECM, in particular IGF-I:VN interactions, in melanoma progression. In addition, given the involvement of TRI complexes in drug resistance^15^, investigating the therapeutic advantages of disrupting such IGF:VN interactions could be beneficial. To date, therapeutic options to treat aggressive melanoma or melanoma with a propensity to metastasize have not been effective. Nonetheless, immunotherapies have significantly improved overall patient survival^20,21^. However, efficient targeted therapeutics, which could be used in combination with such immunotherapies, are required to overcome primary resistance. We have designed peptide-antagonists that are capable of disrupting growth factor:ECM interactions, reducing activation of signaling cascades downstream of IGF-I and providing anti-tumor effects in *in vitro* breast cancer models^12^. The efficacy of such peptide antagonists on melanoma cell function is explored herein. Our proof-of-concept study demonstrates that targeting IGF:ECM molecular interactions may be a viable option for inhibiting processes facilitating the dissemination of melanoma cells.

## Results

### Substrate-bound IGF-I:IGFBP-3:VN complex stimulates melanoma cell proliferation and migration

IGF-I:IGFBP-3:VN trimeric (TRI) complex-stimulated melanoma cell proliferation and migration was assessed using the MTS cell proliferation and Transwell migration assays^12,15^. Cell proliferation was assessed after 72 hours, whereas cell migration was assessed after 15 hours.

**Figure 1:**
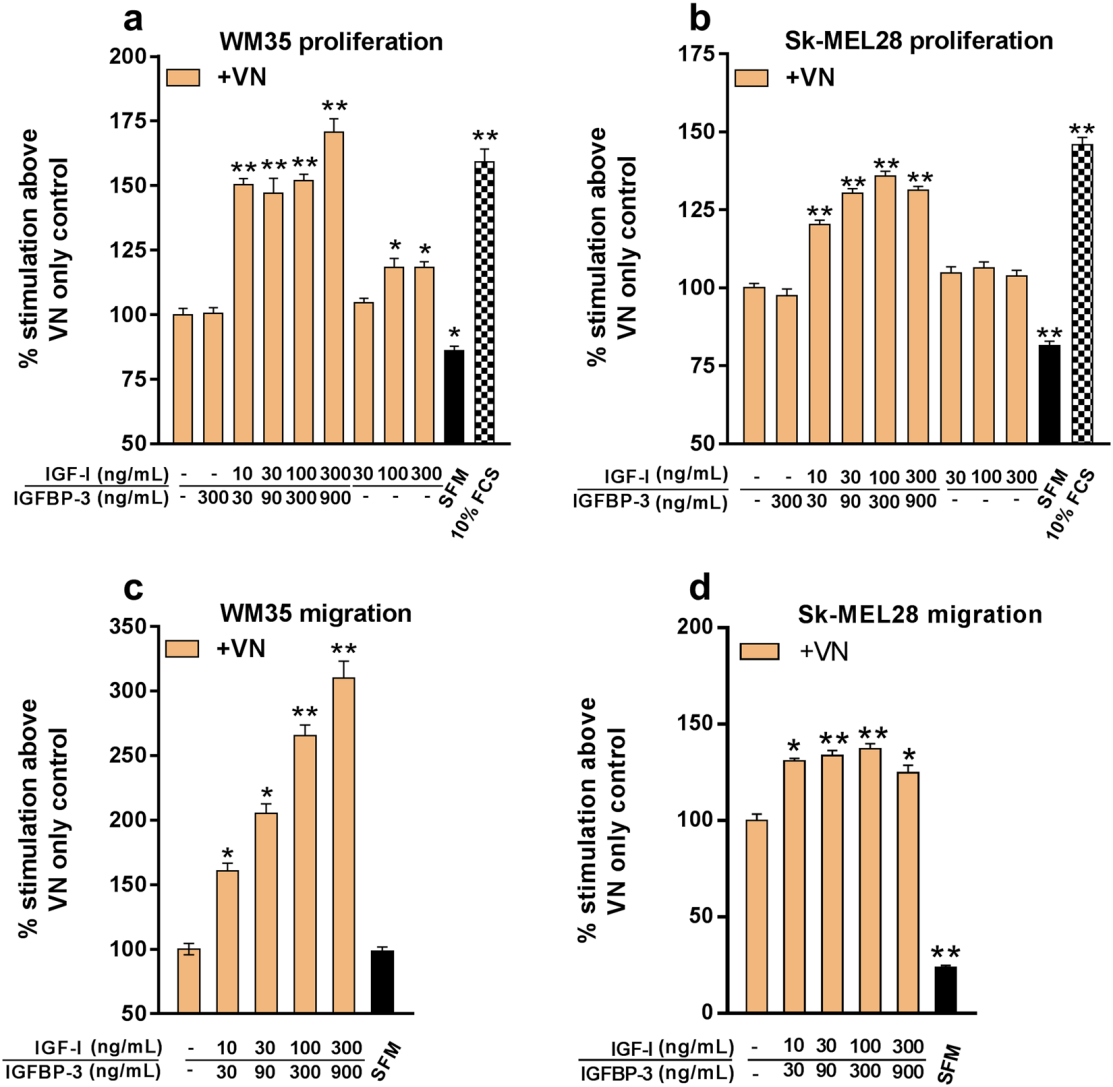
Substrate-bound IGF-I:IGFBP-3:VN complex stimulates melanoma cell proliferation and migration. Proliferation**:** Serum-starved WM35 (**a**) and Sk-MEL28 (**b**) cells were seeded onto 96-well plates pre-coated with VN and combinations of IGF-I and IGFBP-3. Data is expressed as percentage of cells proliferating compared to VN after 72 hours. n = 2 (tested in four wells in each experiment). Migration**:** Serum-starved WM35 (**c**) and Sk-MEL28 (**d**) cells were seeded in the top chamber and allowed to migrate towards VN/IGF-I/IGFBP-3-coated bottom chamber. Data is expressed as percentage of cells migrated compared to VN after 15 hours. Asterisks indicate treatments with significant differences compared to the VN (*p < 0.05, **p < 0.01). SFM = serum-free growth medium. 10% FCS = growth medium containing 10% FCS. Error bars indicate SD.

Treatment of both WM35 and Sk-MEL28 cells with substrate-bound TRI significantly enhanced (*p* < 0.05) cell proliferation in a dose dependent manner compared to VN alone [Fig. 1(a,b)]. The VN and the IGFBP-3 + VN treatment-induced WM35 and Sk-MEL-28 cell proliferation were non-significant to each other. Only marginal increases in proliferation were observed in the VN + IGF-I treatments (without IGFBP-3) in WM35 cells, owing to IGF-I being unable to bind to VN in the absence of IGFBP-3, hence being removed during the washing steps.

In terms of cell migration, the TRI complex significantly enhanced (*p* < 0.05) WM35 cell migration in a dose dependent manner compared to the VN control [Fig. 1(c)]. WM35 cell migration induced by VN was low and similar to that observed in the SFM controls. Additional treatments with increasing doses of IGF-I alone (without VN) did not stimulate migration beyond responses observed with SFM [Fig. S1]).

In Sk-MEL28 cells, however, the basal level of cell migration induced by VN was more pronounced and remained significantly higher (*p* < 0.01) than the SFM control [Fig. 1(d)]. In spite of the high basal level of migration induced by VN, the addition of IGF-I + IGFBP-3 induced a further increase in Sk-MEL28 cell migration, albeit lesser than that induced in WM35 cells. Taken together, the TRI complex is a potent stimulator of melanoma cell proliferation and migration.

### Effect of the TRI complex on melanoma cells cultured in 3D matrices

**Figure 2:**
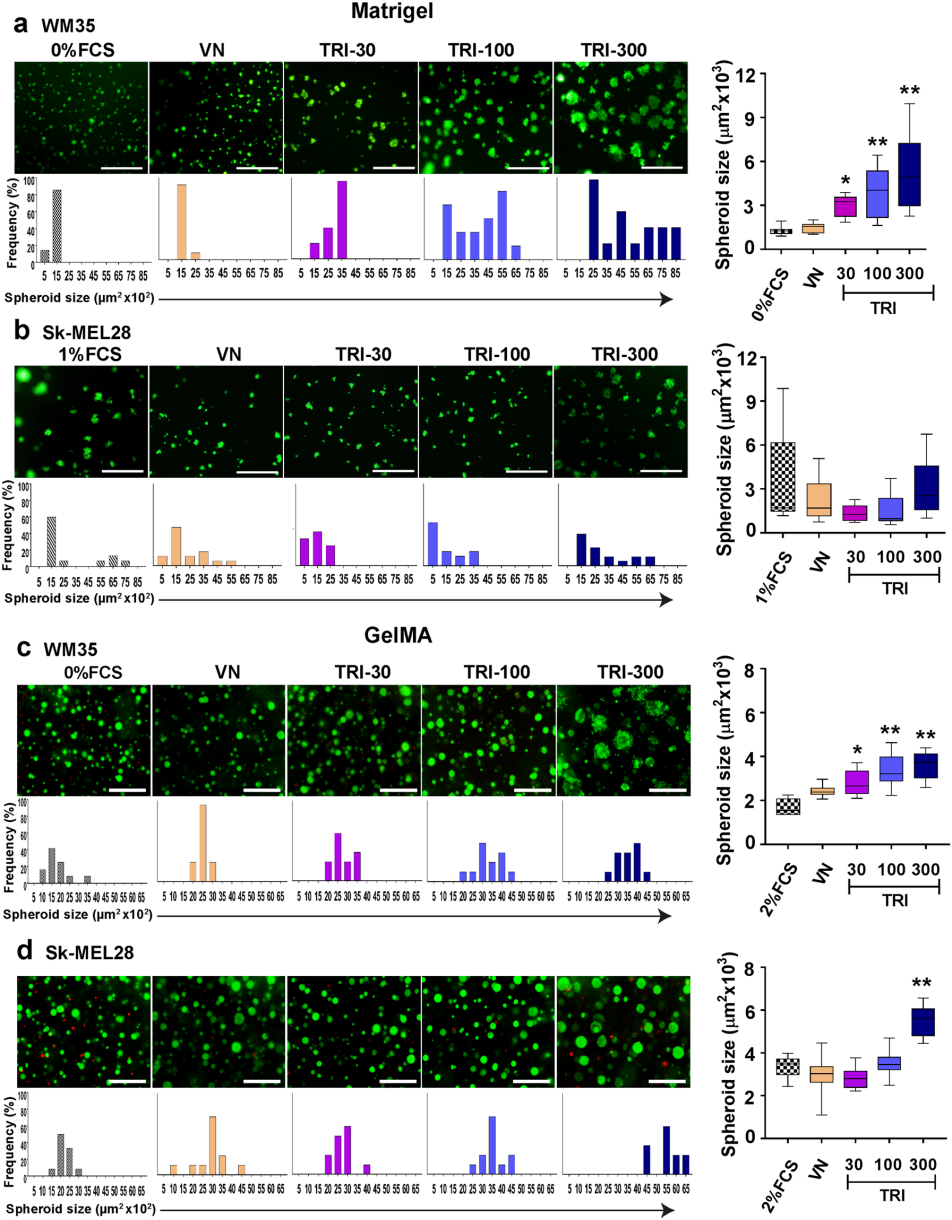
IGF-I:IGFBP-3:VN complex stimulates growth of melanoma spheroids in 3D cultures. WM35 and Sk-MEL28 cells were seeded onto 100% Matrigel™ (**a,b**) or encapsulated in GelMA hydrogel (**c,d**). On day 10 (Matrigel) or day 21 (GelMA), cells were stained with FDA/PI. TRI 30 = 1 ng/mL VN + 30 ng/mL IGF-I + 90 ng/mL IGFBP-3; TRI 100 = 1 ug/mL VN + 100 ng/mL IGF-I + 300 ng/mL IGFBP-3; TRI 300 = 1 μg/mL VN + 300 ng/mL IGF-I + 900 ng/mL IGFBP-3. Bar graphs represent frequency of spheroid sizes. n = 2 (tested in three wells in each experiment). Asterisks indicate treatments significantly different to VN (*p < 0.05, **p < 0.01). 0% FCS = 3D growth medium without serum. Scale bars indicate 100 μm (matrigel) and 200 μm (GelMA). Error bars indicate SD.

Two different 3D systems mimicking the *in vivo* tumor microenvironment (Matrigel^TM^ and gelatin methacryloyl; GelMA)^22,23^ were used to test the effects of the TRI complex on melanoma cells. In both Matrigel^™^ and GelMA, the non-malignant WM35 cells formed larger spheroids in the presence of the TRI complex, as compared to VN alone [Fig. 2(a,c)]. Size quantification of Sk-MEL28 spheroids in Matrigel^TM^, revealed non-significant differences between the tr

eatments [Fig. 2(b)]. However, Sk-MEL28 cells grown in GelMA demonstrated a significant increase in spheroid growth at the highest dose of the TRI complex [Fig. 2(d)]. Taken together, the Matrigel^TM^ and GelMA results suggest that the TRI complex stimulates WM35 and Sk-MEL28 cell proliferation in a 3D microenvironment.

### TRI complex stimulates activation of the IGF-IR and the AKT/ERK cell signaling pathways

In order to understand the mechanisms underlying the TRI complex-induced proliferation and migration, the activation of IGF-IR and its key downstream signaling intermediates ERK1/2 and AKT were investigated.

**Figure 3:**
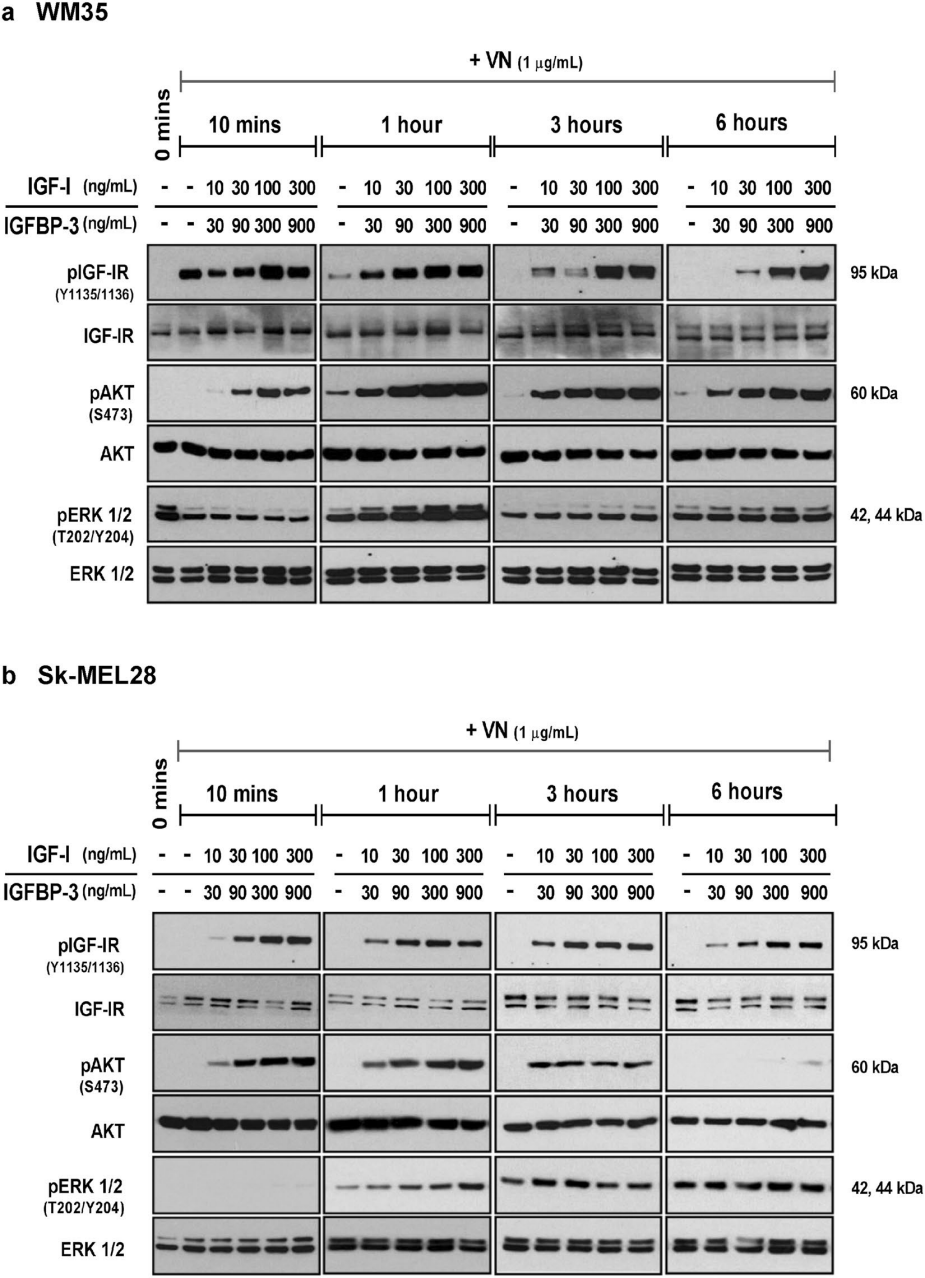
Activation of the IGF-IR, AKT and ERK1/2 signaling intermediates. Total protein from WM35 (**a**) and Sk-MEL28 (**b**) cells exposed to VN, either alone or in combination with increasing concentrations of IGF-I and IGFBP-3, was collected at the indicated time points and assessed by Western blot. Blots were probed for phosphorylated IGF-IR, AKT and ERK1/2 and subsequently stripped and re-probed to determine total levels of IGF-IR, AKT and ERK1/2.

In WM35 cells the TRI complex exclusively induced activation of the IGF-IR across all time points tested [Figs 3(a) and S2(a)]. The TRI complex induced a dose-dependent and sustained activation of AKT, whereas minimal activation of AKT was observed with the VN control [Fig. 3(a)]. In WM35 cells, ERK1/2 phosphorylation was detected across all time points tested, even at 0 minutes (no treatment) [Fig. 3(a)]. This indicates that ERK1/2 was activated in WM35 cells, irrespective of the stimulus. Nevertheless, the TRI complex stimulated ERK1/2 activation above VN at the 1-hour time point.

The IGF-IR and AKT activation profile in Sk-MEL28 cells was similar to that observed in WM35 cells [Figs 3(b) and S2(b)]. In contrast to WM35 cells, ERK1/2 in Sk-MEL28 cells remained un-phosphorylated at 0 minutes (no treatment control), as well as the 10 minute time point. The presence of VN within the TRI complex seemed to be a major contributor to the ERK1/2 activation observed [Fig. 3(b)], as phosphorylated ERK1/2 was detected at 1, 3 and 6 hours, not only in response to the TRI complex, but also to the VN only treatment. Nevertheless at 1 hour, TRI-stimulated activation of ERK1/2 was above VN control. We omitted VN + IGF-I treatments (without IGFBP-3) as these were demonstrated in previous publications^12,15,16^ to not induce activation of IGF-IR, AKT and ERK in the absence of IGFBP-3, due to minimum IGF-I remaining in the wells in the absence of IGFBP-3^15,16^.

### IGFBP-3:VN complexes are present in malignant skin tissue sections

**Figure 4:**
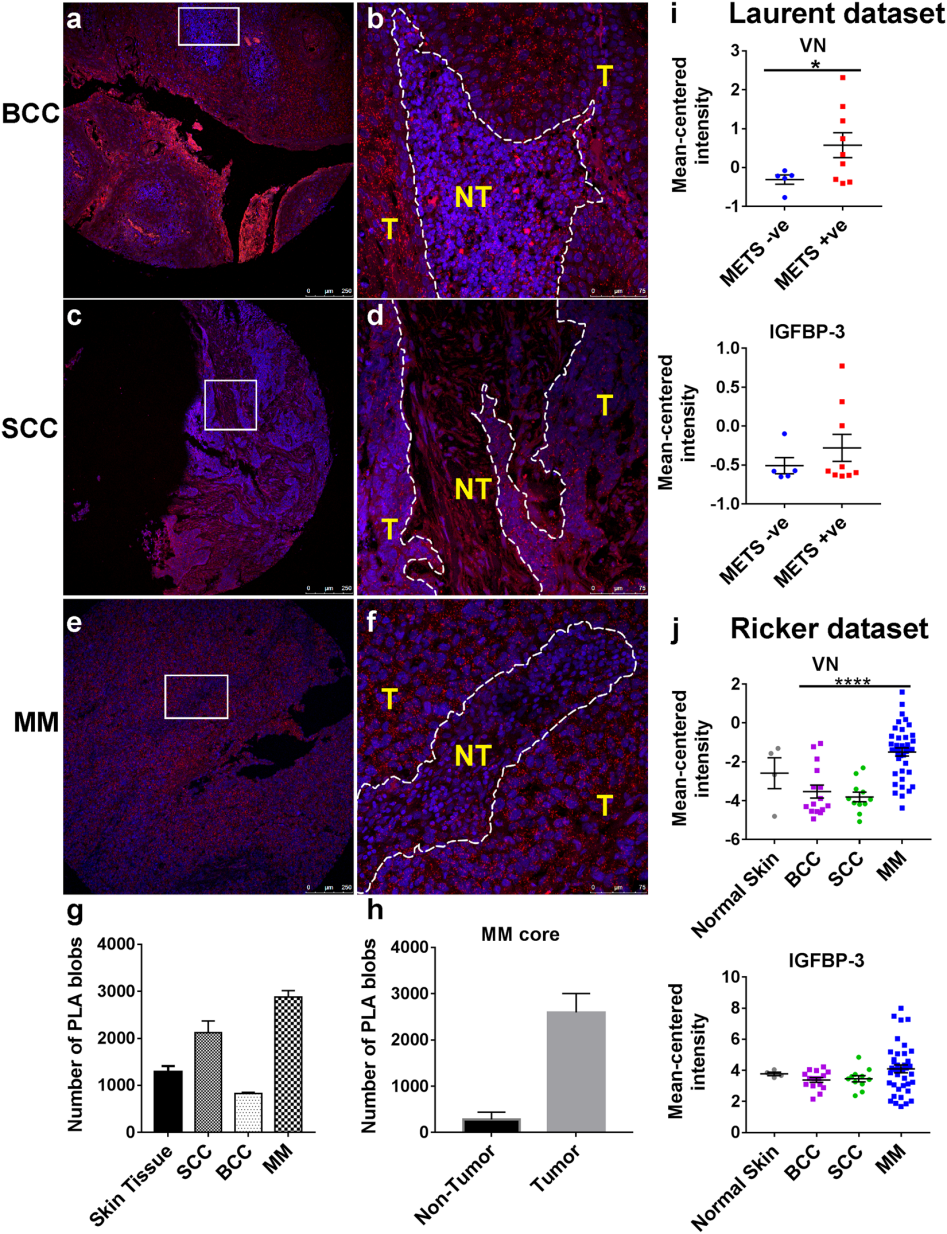
Detection of IGFBP-3:VN complex in FFPE skin cancer TMAs using *in situ* PLA. The VN:IGFBP-3 complexes are observed as bright red dots in: basal cell carcinoma (BCC) core (**a,b**); squamous cell carcinoma (SCC) core (**c,d**); and malignant melanoma (MM) core (**e,f**). The IGFBP-3:VN interaction transcript was visualized with a Cy3-labeled detection oligonucleotide (red), and nuclei are in blue. Tumor regions (T) are demarcated from non-tumor (NT) regions by white dotted lines (**b,d,f**). Scale bars indicate 250 μm and 75 μm. The number of PLA blobs was quantified for each tumor type and normal skin (**g**) and between non-tumor and tumor areas of the MM core (**h**). Clinical datasets from oncomine were used to quantify VN and IGFBP-3 expression in metastatic and non-metastatic tumors (**i**) and between normal and malignant skin sections (**j**).

IGF-I, VN and IGFBP-3 have been individually found within the skin epidermis and dermis, however, their presence together in a complex has not been explored in normal and malignant skin tissues. Therefore, IGFBP-3:VN complexes were investigated using the *in situ* proximity ligation assay (PLA) in normal skin, squamous cell carcinoma (SCC), basal cell carcinoma (BCC) and malignant melanoma (MM) tissue sections. In normal skin tissue IGFBP-3:VN complexes (red PLA “blobs”) were localized in the keratinocyte-rich epidermis [Fig. S3]. The SCC [Fig. 4(c,d)] and MM [Fig. 4(e,f)] sections displayed a higher density of the IGFBP-3:VN PLA “blobs” compared to the BCC sections [Fig. 4(a,b)]. Upon quantification of the PLA signal MM had the highest density of PLA “blobs” and BCC with the least [Fig. 4(g)]. Figure S6 outlines the quantification process. Interestingly, the IGFBP-3:VN PLA “blobs” were predominantly tumor cell associated compared to the non-tumor regions of MM cores [Fig. 4(h)]. These results provide the first identification of IGFBP:VN complexes in skin cancer and melanoma tissues. We further investigated VN and IGFBP-3 expression in publically available clinical datasets^24,25^. Mean-centered signal intensity for IGFBP-3 and VN were calculated in metastasis positive and negative patients (Laurent dataset), and across SCC, BCC and MM (Riker dataset). While no significant changes in expression of IGFBP-3 were detected, VN expression was higher in metastatic tumors [Fig. 4(i)]. Interestingly, expression of VN was also significantly higher in tumors of MM compared to BCC and SCC [Fig. 4(j)]; supporting our PLA data. It was therefore of interest to explore therapeutic strategies focused on disrupting these IGFBP:VN complexes.

### Peptide antagonist disrupts TRI complex-stimulated cell migration and tumor spheroid growth

**Figure 5:**
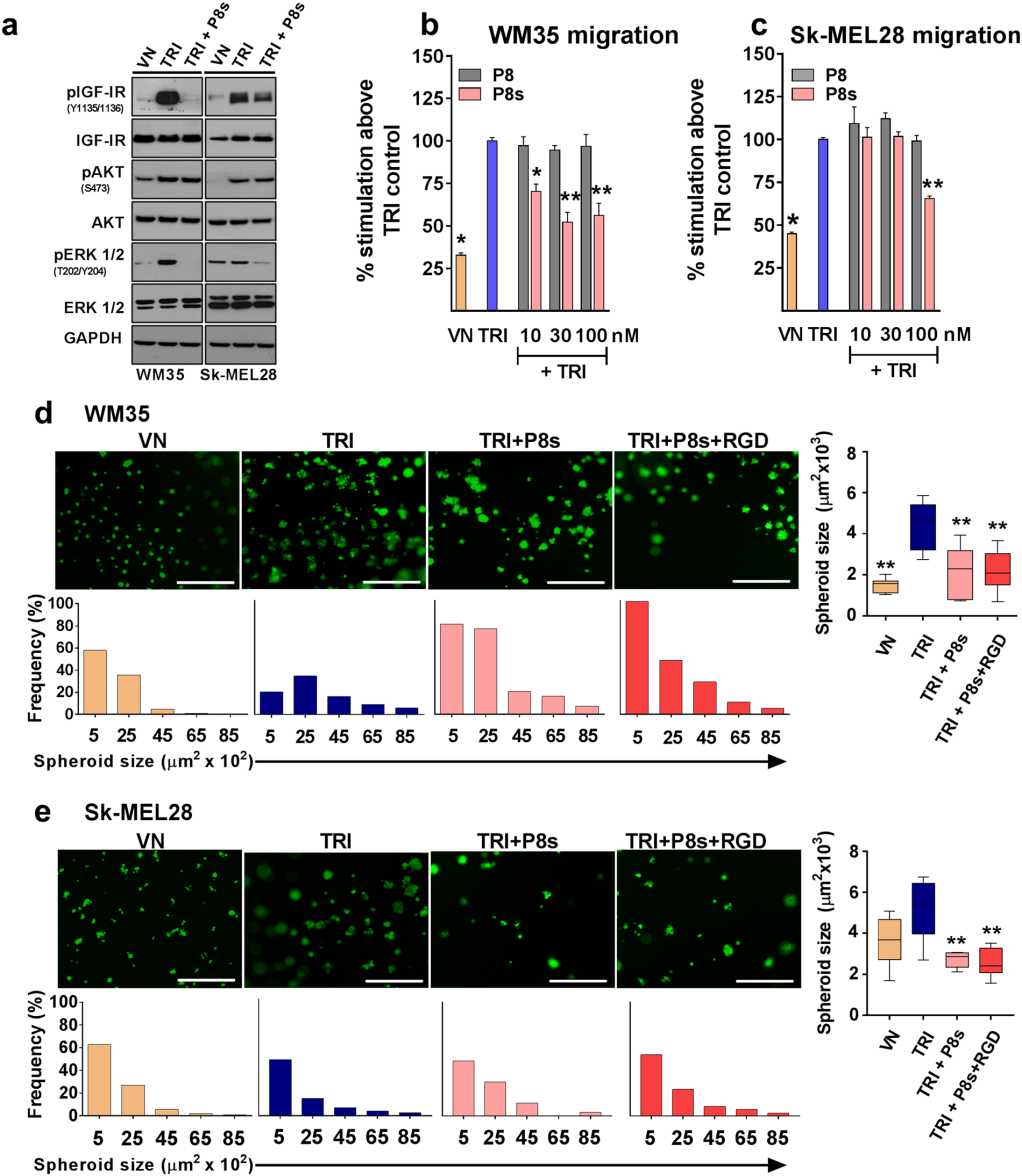
Effect of the peptide P8s on TRI-stimulated signalling, cell migration and spheroid growth. Western blot: Cell lysates were collected after 1 hour and levels of phosphorylated and total IGF-IR, AKT, and ERK1/2 were determined by Western blot analysis (**a**). Migration: Serum-starved WM35 (**b**) and Sk-MEL28 (**c**) cells were allowed to migrate for 15 hours. Data is expressed as percentage of cells that migrated compared to TRI control. Matrigel^**TM**^**:** WM35 (**e**) and Sk-MEL28 (**f**) cells were seeded onto 100% Matrigel^™^ and subsequently treated with VN or TRI supplemented with P8s. TRI = 1 μg/mL VN + 300 ng/mL IGF-I + 900 ng/mL IGFBP-3. n = 2 (tested in three wells in each experiment). Asterisks indicate treatments with significant differences compared to the TRI control (*p < 0.05, **p < 0.01). Scale bars indicate 100 μm. Error bars indicate SD.

We have previously outlined the design and functional relevance of peptide antagonists (termed P8) targeting the TRI complex. By disrupting the association between IGFBP-3 and VN, the peptide prevents IGF-I from binding to VN and thereby inhibits the downstream effects of IGF-I^12^. Here we generated a shorter version of P8, termed P8s (sequence: RRVDTV). P8s and P8 had similar ability to disrupt the TRI complex [Fig. S4]. Addition of the P8s peptide substantially reduced the TRI complex-induced activation of the IGF-IR and ERK1/2 phosphorylation [Fig. 5(a)] in both WM35 and Sk-MEL28 cells. However, P8s did not alter TRI-induced activation of AKT in both cell lines. In summary, peptide P8s interferes with TRI complex-induced IGF-IR and ERK1/2 activation in both the WM35 and Sk-MEL28 melanoma cells.

Transwell migration assays were performed in the presence of increasing doses of the P8s peptide. The larger P8 peptide^12^ was also included for comparative purposes. In both WM35 and Sk-MEL28 cells, the larger P8 peptide had no inhibitory effect on TRI complex-stimulated migration [Fig. 5(b,c)]. In contrast, the smaller P8s peptide significantly reduced (*p* < 0.05) the TRI complex-induced migration of WM35 and SK-Mel28 cells [Fig. 5(b)].

The ability of the P8s peptide to inhibit tumor-spheroid growth in 3D Matrigel™ culture was further investigated. When WM35 or Sk-MEL28 cells were grown in the presence of the TRI complex + P8s peptide there was a decrease in spheroid size compared to the TRI complex control. ImageJ analysis revealed that cells treated with TRI + P8s and TRI + P8s + RGD had fewer spheroids, and they were of smaller size compared to cells grown in the presence of TRI alone [Fig. 5(d,e)]. These data suggest that P8s can competitively disrupt the TRI complex and inhibit IGF-I-induced cell migration and spheroid formation of melanoma cells. Co-treatment of P8s (IGF-I targeting) and RGD peptide (VN-targeting) did not result in any additive effect in both 2D [Fig. S7] and 3D assays [Fig. 5(d,e)].

In summary our *in vitro* and *ex vivo* data provide evidence for the role of IGF-I: matrix protein interactions in cell growth and migration. In addition, peptides that disrupt growth factor:ECM interactions appear promising for targeting growth factor-induced melanoma progression.

## Discussion

Experimental evidence strongly suggests growth factor:ECM interactions play critical roles in promoting a variety of skin diseases^6,26^. *In vivo* it can regulate growth factor diffusion rates, facilitate the storage of growth factors in a readily available form, and modulate growth factor:receptor interactions^27,28^. We have previously demonstrated that IGF-I associates with the ECM glycoprotein VN, through IGFBP-2, -3, -4 and -5, to form IGF-I:IGFBP:VN (TRI) complexes^11^. Such TRI complexes were found to enhance cellular functions in human keratinocytes^14,29^, and normal as well as malignant breast cells^12,15,16^. These effects were mediated through VN and IGF-I co-operation where IGFBP-bound IGFs enhance signalling by activating integrin/IGF-IR cross-talk, followed by the recruitment of the PI3-K/AKT and the ERK/MAPK pathway^15,16^.

Our study investigated the consequence of IGF-I:IGFBP:VN interactions on melanoma cell function and provides insights into a new therapeutic approach that targets the formation of such multiprotein complexes. *In vivo*, sequestered delivery from the matrix is known to occur for many growth factors, including IGF-I. There is significant evidence that growth factors and ECM proteins interact^29^, facilitating local storage of growth factors^30^ in a “bioavailable” form. This localizes ligands in close proximity to their cognate cell surface receptors, thereby prolonging the activation of downstream signaling cascades^31^, and in turn, enhancing growth factor-induced biological functions. In melanoma all three components of the IGF-I:IGFBP:VN complex are independently shown to be associated with different stages of the disease^32–34^, wherein IGF-I:IGFBP-3 would more likely be associated with ECM proteins like VN and not present in “solution”. Hence, to mimic this *in vivo* situation, we adopted a substrate substrate-bound approach, which better demonstrates the influence of growth factors and ECM interactions on tumor cell properties. Using substrate-bound 2D assays, the TRI complex was demonstrated to be a potent stimulator of proliferation and migration in both WM35 and Sk-MEL28 melanoma cells [Fig. 1]. Since the dimeric IGFBP-3:VN complex stimulated similar responses to that found with VN alone, it suggests IGF-I was the predominant effector of TRI complex-induced cell proliferation. Unlike early-stage melanoma, late stage melanoma has been shown to be largely growth factor independent^34^. Of note, VN ligation to its key integrin receptors, particularly α_v_β_3_, which is highly expressed in Sk-MEL28 cells^31^, has been shown to activate IGF-IR signaling independent of IGF-I^10,35^. This may explain why the metastatic Sk-MEL28 cells were only weakly responsive to the TRI complex compared to early-stage WM35 cells.

*In vitro* 3D models, like Matrigel and GelMA^22,36^, better recapitulate the *in vivo* tumor microenvironment, wherein their ECM can facilitate growth factor:ECM protein interactions^37,38^. Both the Sk-Mel28 and WM35 melanoma cells formed metabolically active spheroids resembling primary tumors [Fig. 2]. Importantly, these long-term assays were conducted using minimal levels of serum (0–2%), providing evidence that the TRI complex is sufficient to support the growth of melanoma cells over prolonged periods. Possibly due to the sustained activation induced by the TRI complex of the PI3K/AKT and ERK1/2 pathways [Figs 3 and S2], known to be activated in response to IGF-I^39–41^ and deregulated in melanoma^42^.

While the individual components of the complex have been studied in tissues, the clinical relevance of the TRI complex has not yet been investigated. Indeed, overexpression of IGFBP-3 is found in advanced melanoma tumors^32,39^. Likewise, serum VN was significantly elevated in patients with melanoma^33,43^, with tumor growth and metastasis shown to also be dependent on the presence of VN in the ECM^44,45^. When assessed for co-expression of VN and IGFBP-3, we found IGFBP-3:VN interactions predominantly in malignant melanoma and SCC patient tumors, and mainly tumor-cell associated [Fig. 4]. However, when assessed individually using publically available gene expression data only VN expression correlated with our *in situ* PLA data [Fig. 4], which suggests that evaluating growth factor:matrix interactions, as opposed to studying them in isolation, may add value to their prognostic and predictive potential. Taken together, growth factor:ECM interactions may play an important role in the progression of cutaneous neoplasia, hence should be considered in the design and the development of growth factor/ECM targeting therapeutics.

Interestingly, the resistance observed to growth factor receptor-targeting therapies may be attributed, at least in part, to growth factor:ECM interactions. For instance, IGF-IR inhibitors such as AVE1642 and R1507 have only been sub-optimal in late-stage clinical trials^46–48^. In such cases growth factor:ECM interactions may trigger additional compensatory cell signaling pathways, including integrin-mediated signaling that may dampen the therapeutic response^8,49^. Thus, there is a need for alternative therapeutic strategies that take into account the role of the ECM in facilitating growth factor responses.

We recently reported the development of peptide-based antagonists capable of disrupting the TRI complex and its downstream effects in IGF-IR expressing breast cancer cells^12^. Interestingly, both WM35 and Sk-Mel28 melanoma cells had a reduced capacity to proliferate and migrate in the presence of the P8s peptide compared to the TRI complex [Figs 5(b,c), S7]. In spite of the IGF-I remaining in the wells during the entire duration of the 3D Matrigel experiment, the peptide was able to dampen IGF-I responses, potentially due to the weak tethering of IGF-I to ECM components (including VN) in the presence of P8s, leading to less IGF-I being available for IGF-IR stimulation. Interestingly, P8 (the longer version of P8s) was less effective compared to P8s [Fig. 5], despite its similar ability to dislodge IGF-I:IGFBP-3 from VN [Fig. S4]. This was also observed in a previous study with another peptide P7, which efficiently disrupted the complex but offered minimal functional effects^12^. Indeed, the impact of the IGFBP-3-binding peptides on IGF-I-independent effects will need to be investigated to fully understand the off-target effects of these peptides. Nevertheless, it is known that residues within P8s bind to IGFBP-3 at sites distinct to IGF-I-binding, thereby maintaining normal IGFBP:IGF-I–binding interactions^12^. Currently, P8s is still being optimized for pre-clinical investigations.

Newer approaches to overcome drug resistance of melanoma cells to MAPK/MEK inhibitors are being investigated. A number of co-targeting approaches have been used^50,51^, however, achieving robust abrogation of this cascade has remained a formidable challenge^52^. We demonstrate here the use of peptide antagonists for inhibition of IGF-I induced MAPK/ERK signaling in BRAF-mutant Sk-MEL28 and WM35 melanoma cells [Figs 5(a) and S5]. However, AKT phosphorylation remained unaffected upon treatment with the peptide. This suggests that AKT compensation may, in part, undermine the effects of the peptides. This is in agreement with study reporting AKT activation upon MAPK/ERK inhibition, leading to *de novo* resistance in BRAF-mutant Sk-MEL28 melanoma cells exposed to epidermal growth factor (EGF)^53^. Recently, Barbara Herkert *et al*. (2016) reported that combinations of AKT + IGF-IR, and MAPK pathway inhibitors is required to achieve maximal response in BRAF-mutant melanoma patients. Together with the findings demonstrated in this study, we propose that IGFBP:VN targeting peptides are a viable therapeutic option and in combination with AKT/PI3K inhibitors could be employed for the treatment of skin malignancies, namely SCC and melanoma, in particular for patients with BRAF-mutant tumors and/or tumors overexpressing components of the IGF:VN axis.

## Materials and Methods

### Proteins and reagents

Plasma purified human VN was from Promega (NSW, Australia). Human recombinant IGF-I and IGFBP-3 were from GroPep (SA, Australia). Bovine Serum Albumin (BSA) Fraction-V was from Life Technologies (NSW, Australia). Monoclonal antibodies for phospho-IGF-I Receptor (Tyr1135/1136) (19H7), phospho-p44/42 MAPK (ERK1/2) (Thr202/Tyr204) (E10), phospho-AKT (Ser473) (D9E) and GAPDH (14C10) were from Cell Signaling Technology (MA, USA). Rabbit anti-IGF-IRβ (C-20) was from Santa Cruz Biotechnology (CA, USA). Mouse anti-VN was from Promega (WI, USA). Rabbit anti-IGFBP-3 antibody was obtained from Robert Baxter (Kolling Institute of Medical Research, Australia). Growth factor-reduced (GFR) phenol red-free Matrigel™ was from BD Biosciences (NJ, USA). The peptides P8 (RVNLRTRRVDTVDPPYPRS-NH2), P8s (RRVDTV-NH2) and RGD (H-CRGDSCG-OH) were synthesized by Mimotopes (VIC, Australia).

### Cell culture

WM35, a radial growth phase cell line, was provided Dr. Nikolas Haass and the malignant SK-Mel28 cell line was provided by Dr. Brian Gabrielli (both University of Queensland, Australia). WM35 cells were maintained in MCDB 153 medium (Sigma-Aldrich) supplemented with 4% FCS, 20% Leibovitz’s medium (Life Technologies), bovine insulin (5 µL/mL), CaCl_2_ (1.68 mM), sodium bicarbonate (7.5% w/v). SK-Mel-28 cells were maintained in RPMI 1640 medium supplemented with 2 mM L-glutamine, 25 mM HEPES, 10% FCS. Both media contained Penicillin (50 U/mL) + Streptomycin (50 µg/mL) and 0.01% Gentamycin (Life Technologies). The identity of both these cell lines was authenticated using short tandem repeat (STR) profiling by CellBank Australia and tested for mycoplasma using the MycoAlert^TM^ mycoplasma detection kit (Lonza).

### Pre-binding of proteins

The pre-binding of proteins to culture ware followed previously published procedures^15,16^. Briefly, culture plates were coated with 1 µg/mL VN in serum-free medium (SFM) and incubated for 3 hours at 37 °C. Unbound VN was aspirated and wells were blocked with 0.5% BSA/SFM. Subsequently, IGF-I and IGFBP-3 suspended in 0.05% BSA/SFM at various concentrations was added to the plates and incubated overnight at 4 °C. For assays using the P8s and P8 peptides, the plates were coated with VN, IGF-I and IGFBP-3 suspended in 0.05% BSA/SFM, with or without P8s/P8 and incubated at 37 °C for 3 hours. In all cases, unbound growth factors were removed with washing prior to seeding the cells. The lower chambers and under-side of 8 µm-pore Transwells were also coated following the same procedure. In all experiments containing peptides, peptide + IGF-I alone controls were not included as these peptides do not bind IGF-I but are designed to bind to VN-binding sites on IGFBP-3.

### MTS cell proliferation assay

The cells were serum-starved for 4 hours and seeded at a density of 5 × 10^3^ cells/well onto 96-well plates pre-bound with the respective protein treatment combinations. The cells were incubated for 72 hours and cellular proliferation was measured using the CellTitre 96^®^ AQueous One Solution Cell Proliferation Assay (Promega). The absorbance was recorded at 490 nm (Benchmark Plus, Bio-Rad).

### Transwell® migration assays

Cell migration was assessed as previously described^13,15,16^. The cells were serum-starved for 4 hours and seeded at a density of 6 × 10^4^ cells/well in the upper chambers of pre-bound Transwells and incubated for 15 hours. Cells that had migrated to the lower surface of the membrane were fixed in 3.7% para-formaldehyde and stained with 0.01% crystal violet. The relative number of migrated cells was assessed by extracting the crystal violet stain in 10 mM acetic acid. The optical density of the extracted stain was determined at 595 nm (Benchmark Plus, BioRad.

### Western blot analysis for protein expression

Cells were serum-starved for 4 hours and 8 × 10^5^ cells/well were seeded in 6-well plates pre-bound with the respective protein treatments. Cells were lysed using radioimmunoprecipitation assay (RIPA) protein lysis buffer containing phosphatase and protease inhibitors (Pierce, Thermo Fisher Scientific). Twenty microgram total protein was separated on SDS-PAGE gels (Life Technologies) under reducing conditions prior to being transferred onto nitrocellulose membranes (Life Technologies). The membranes were probed with antibodies and visualized using the enhanced chemiluminescence (ECL) system (Amersham Biosciences).

### 3D Matrigel™ assay

Optical-bottom 96-well plates were coated with 100% growth-factor-reduced-Matrigel™. Using the 3D On-Top method^54^, 1 × 10^3^ cells/well cells were suspended in 3D growth medium (2.5% Matrigel™ +/− 1% FCS). The cells were incubated for two days to allow spheroid formation, following which 3D growth medium containing VN, IGF-I and IGFBP-3 +/− peptides P8s or RGD were added to the wells and replaced every two days for up to ten days. Live cells were stained using fluorescein diacetate (FDA) dye and imaged using fluorescence microscopy (Nikon Eclipse TS) and analyzed using ImageJ (Supplementary Methods).

### 3D GelMA assay

GelMA was synthesized following previously published protocols^22^. To encapsulate cells, GelMA was dissolved in PBS containing 0.05% (w/v) 1-[4-(2-hydroxyethoxy)-phenyl]-2-hydroxy-2-methyl-1-propanone (Irgacure 2959; BASF; Ludwigshafen, Germany) at 37 °C. Sk-MEL28 and WM35 cells were suspended in the hydrogel precursor solution at a density of 2 × 10^5^ cells/mL (6,400 cells per gel), transferred to a custom casting mold to produce hydrogel constructs with the dimension of 4 mm × 4 mm × 2 mm, and then photocrosslinked by exposure to 365 nm light at an approximate intensity of 2.6 mW/cm^2^ in a CL-1000 crosslinker (UVP; CA, USA) for 15 min. The hydrogel constructs were cultured in stripped green’s medium with 2% FCS containing VN, IGF-I and IGFBP-3 +/− P8s peptide at 37 °C, 5% CO_2_ for the duration of the experiment.

### Proximity ligation assay

Human tissue microarrays (TMA) incorporating 10 cases of skin tumor and 2 non-neoplastic tissues, in duplicates, were purchased from US Biomax Inc. (Cat# SK242). The TMA information is outlined in Figure S8. The TMA slides were deparaffinised and subjected to antigen retrieval for 20 mins at 98 °C in 10 mM Tris-EDTA (pH 9). The IGFBP-3:VN interactions were detected with *in situ* PLA, performed as published^12,55^ using anti-VN (1:200) and anti-IGFBP-3 (1:200) antibodies. The tissue sections were incubated with secondary antibodies conjugated with oligonucleotide probes (Olink Bioscience). The IGFBP-3:VN transcript was detected using Cy3-labelled detection probes. The PLA signals were analyzed with confocal microscopy and quantified using ImageJ (Supplementary Methods).

### Data availability

The Laurent and Ricker datasets analysed during the current study are available in the Oncomine repository, www.oncomine.com. All other data generated in this study including supplementary information are included in this published article.

## Electronic supplementary material

Supplementary figures and methods
