# Supplementary material for: Targeting Insulin-Like Growth Factor-I and Extracellular Matrix Interactions in Melanoma Progression
Source: Sci Rep. 2018 Jan 12;8:583. doi: 10.1038/s41598-017-19073-4 (PMC5766529; doi:10.1038/s41598-017-19073-4)
Supplement: Supplementary file 1 — Supplementary figures and methods [file 41598_2017_19073_MOESM1_ESM.pdf]

TARGETING INSULIN-LIKE GROWTH FACTOR-I AND EXTRACELLULAR MATRIX INTERACTIONS IN MELANOMA PROGRESSION

Berline Murekatete[1*], Ali Shokoohmand[1,2*], Jacqui McGovern[1], Lipsa Mohanty[1] Christoph Meinert[1], Brett G Hollier[2], Alfred Zippelius[4], Zee Upton[1,3] and Abhishek S Kashyap[1,4*]

Supplementary Figures

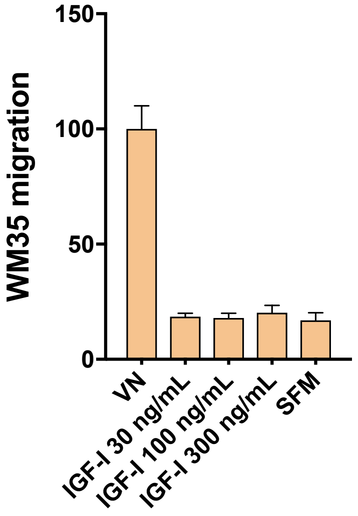

Supplementary Figure S1: IGF-I alone does not stimulate melanoma cell migration in substrate-bound setup.

Serum-starved WM35 cells were seeded in the top chamber and allowed to migrate towards bottom chamber of the Transwell insert pre-coated in a substrate-bound setup with VN (1 µg/mL) or IGF-I at indicated concentrations. Serum-free medium (SFM) was used as control. Data is obtained from two replicate experiments, with each treatment tested in triplicates. Data is expressed as percentage of cells migrated compared to VN after 15 hours. Error bars indicate SD.

a WM35

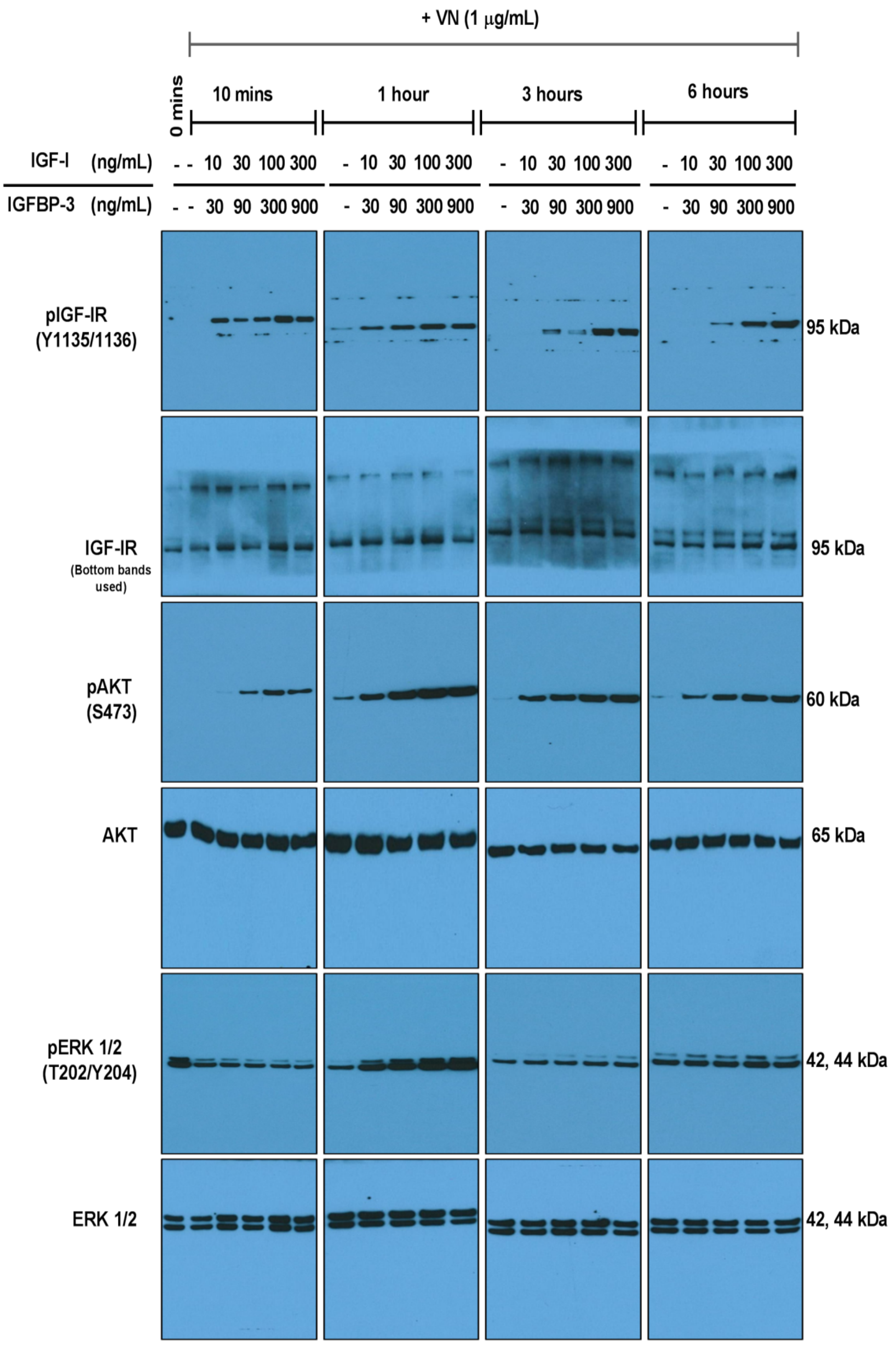
+ VN (1 μg/mL)
0 mins
10 mins
1 hour
3 hours
6 hours
IGF-I (ng/mL) - - 10 30 100 300 - 10 30 100 300 - 10 30 100 300 - 10 30 100 300
IGFBP-3 (ng/mL) - - 30 90 300 900 - 30 90 300 900 - 30 90 300 900 - 30 90 300 900
pIGF-IR (Y1135/1136)
95 kDa
IGF-IR (Bottom bands used)
95 kDa
pAKT (S473)
60 kDa
AKT
65 kDa
pERK 1/2 (T202/Y204)
42, 44 kDa
ERK 1/2
42, 44 kDa

b Sk-MEL28

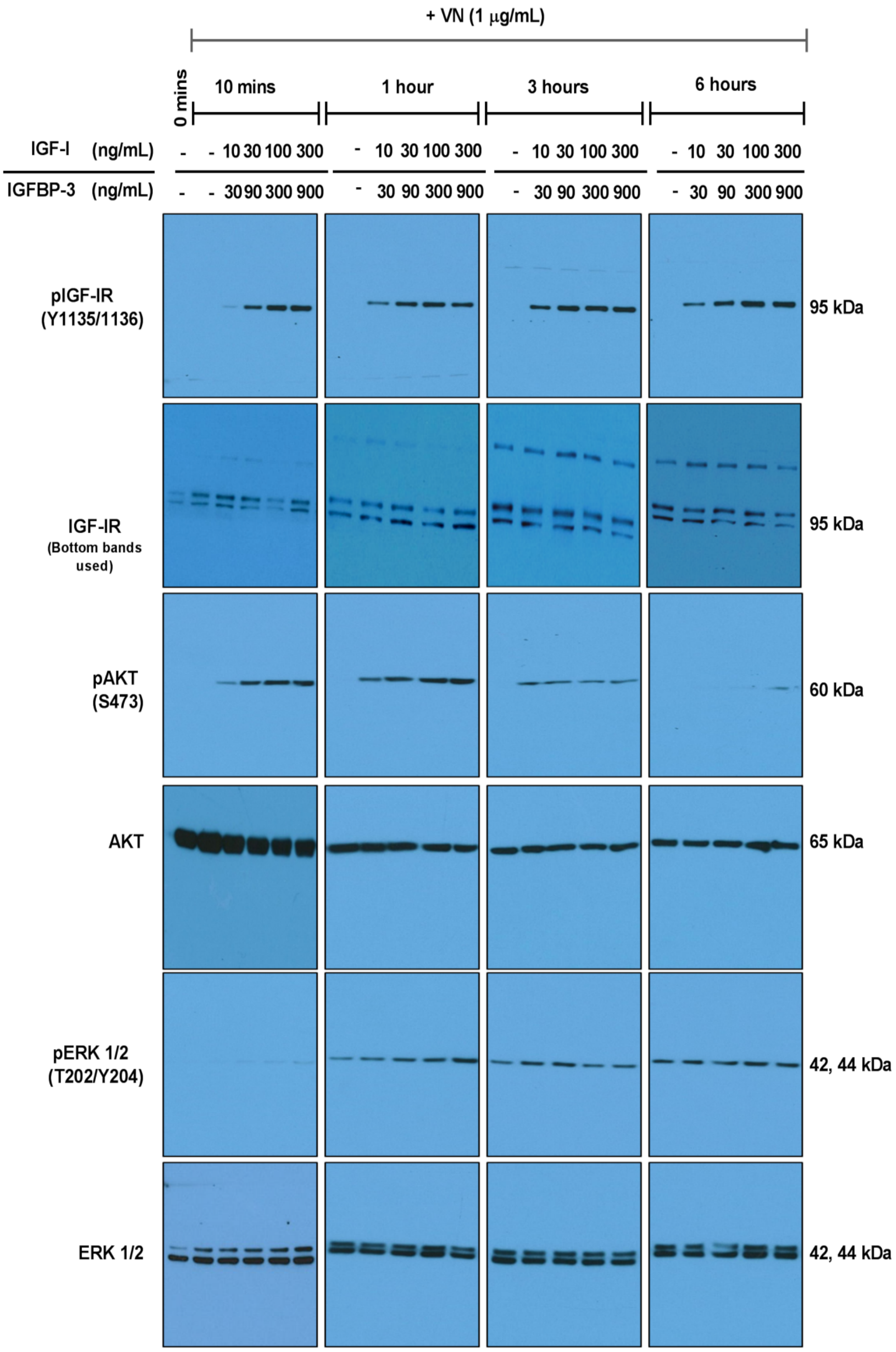

Supplementary Figure S2: Activation of the IGF-IR, AKT and ERK1/2 signaling intermediates (extended blots).

Total protein from WM35 (**a**) and Sk-MEL28 (**b**) cells exposed to VN, either alone or in combination with increasing concentrations of IGF-I and IGFBP-3, was collected at

the indicated time points and assessed by Western blot. Blots were probed for phosphorylated IGF-IR, AKT and ERK1/2 and subsequently stripped and re-probed to determine total levels of IGF-IR, AKT and ERK1/2.

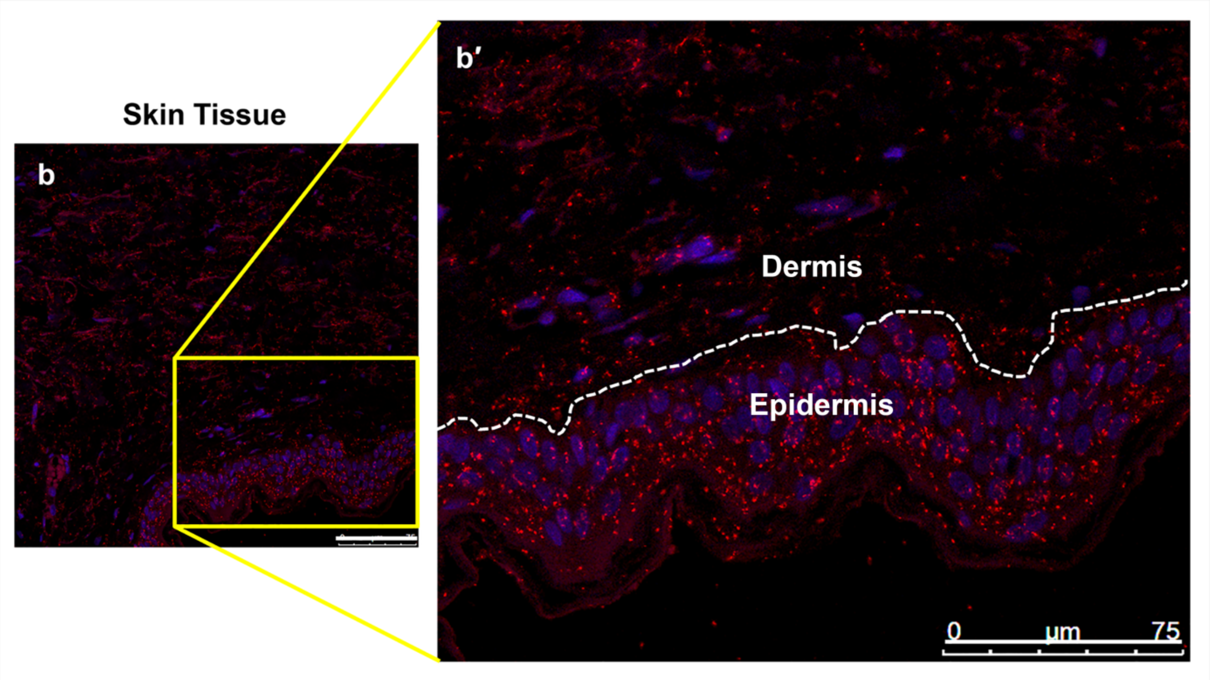

Supplementary Figure S3: Normal skin VN:IGFBP-3 transcript

TMA core of normal skin tissue was stained for VN:IGFBP-3 complex using *in situ* PLA. Epidermis and dermis are demarcated with dotted line.

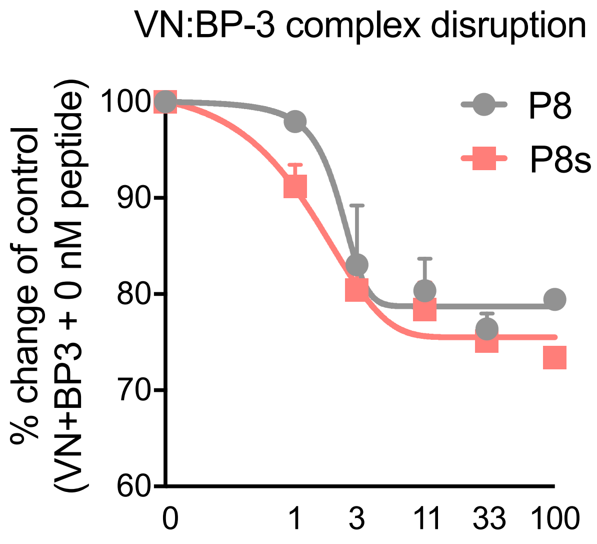

Supplementary Figure S4: IGFBP-3 release from VN upon co-incubation with peptides P and P8s.

The amount of IGFBP-3 remaining in VN-coated wells after increasing concentration of P8 or P8s peptide treatments was assessed using anti-IGFBP-3 antibody. Data were curve-fit using the non-linear log(inhibitor) vs the response model in Graph-Pad prism. Data is obtained from three independent replicates. Error bars indicated SD.

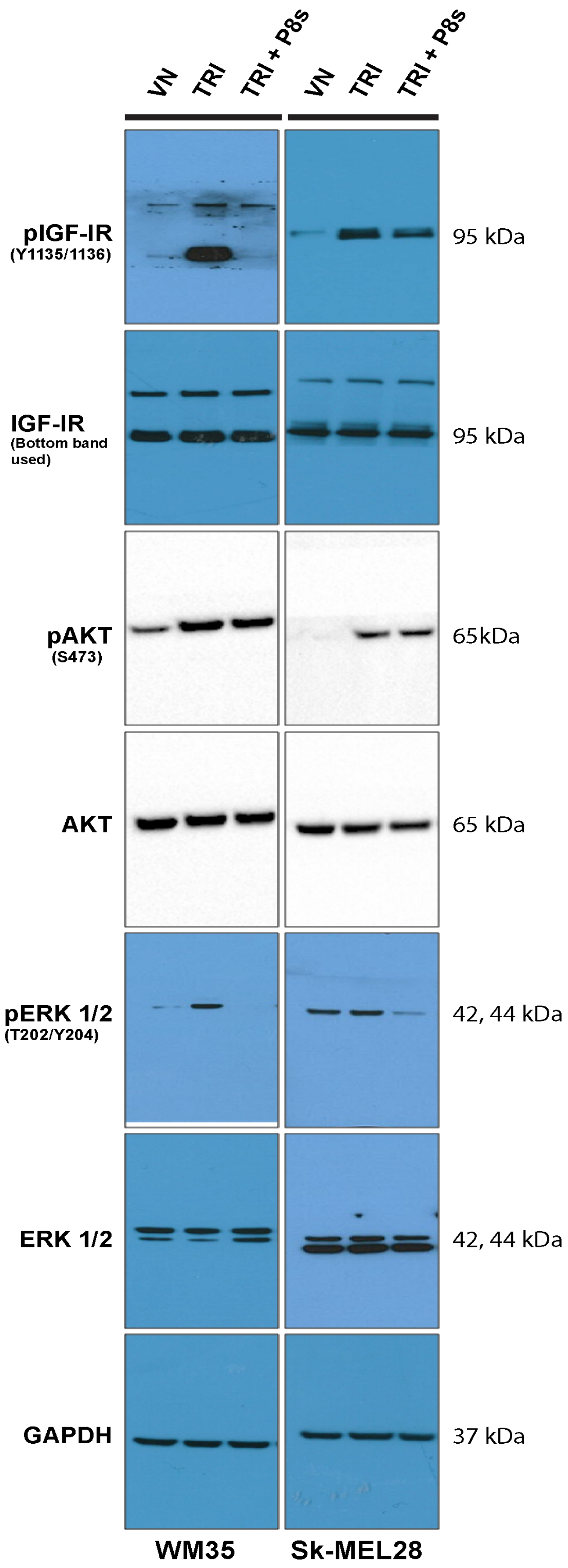
VN
TRI
TRI + P8s
VN
TRI
TRI + P8s
pIGF-IR
(Y1135/1136)
95 kDa
IGF-IR
(Bottom band used)
95 kDa
pAKT
(S473)
65kDa
AKT
65 kDa
pERK 1/2
(T202/Y204)
42, 44 kDa
ERK 1/2
42, 44 kDa
GAPDH
37 kDa
WM35
Sk-MEL28

Supplementary Figure S5: Effect of the peptide P8s on TRI-stimulated signalling, cell migration and spheroid growth (extended blots).

Cell lysates were collected after 1 hour and levels of phosphorylated and total IGF-IR, AKT, and ERK1/2 were determined by Western blot analysis

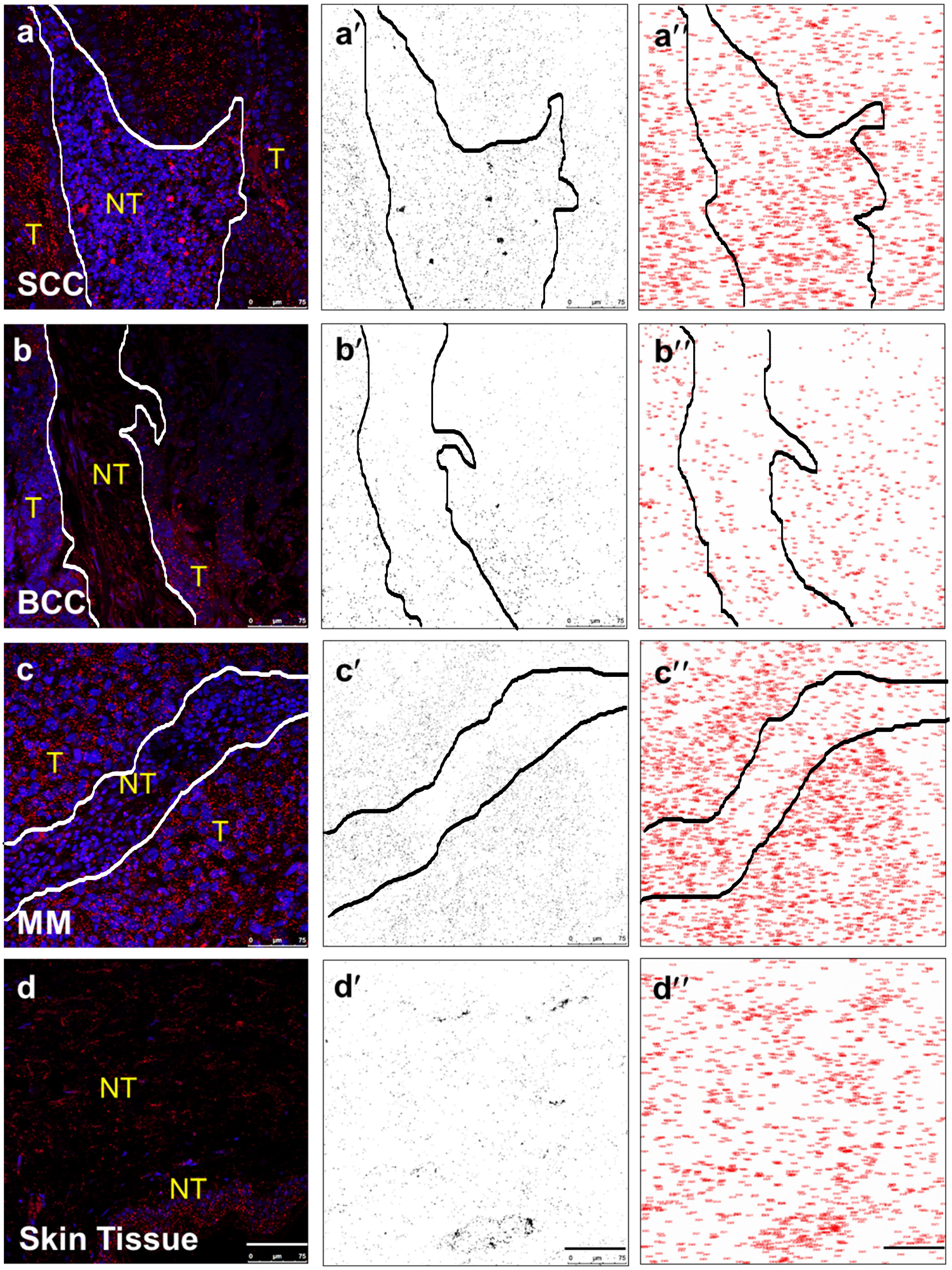

Supplementary Figure S6: Quantification of PLA "blobs"

Maximum-intensity projection images at ×40 objectives for *in situ* PLA staining of VN:IGFBP-3 transcript in FFPE TMAs are observed as red dots in squamous cell

carcinoma (SCC) core (**a**), basal cell carcinoma (BCC) core (**b**), malignant melanoma (MM) core (**c**) and adjacent skin tissue core (**d**). Quantification of PLA "blobs" on exemplary tissue area with non-tumor (NT) and tumor (T) bulk shown as indicated (dotted line). ImageJ analysis and quantification of PLA signals indicate intensity in black dots (a′-d′) and red numbers represents number of VN:IGFBP-3 transcripts in the cores (a′′-d′′). Scale bar is 75 μm as indicated on the images.

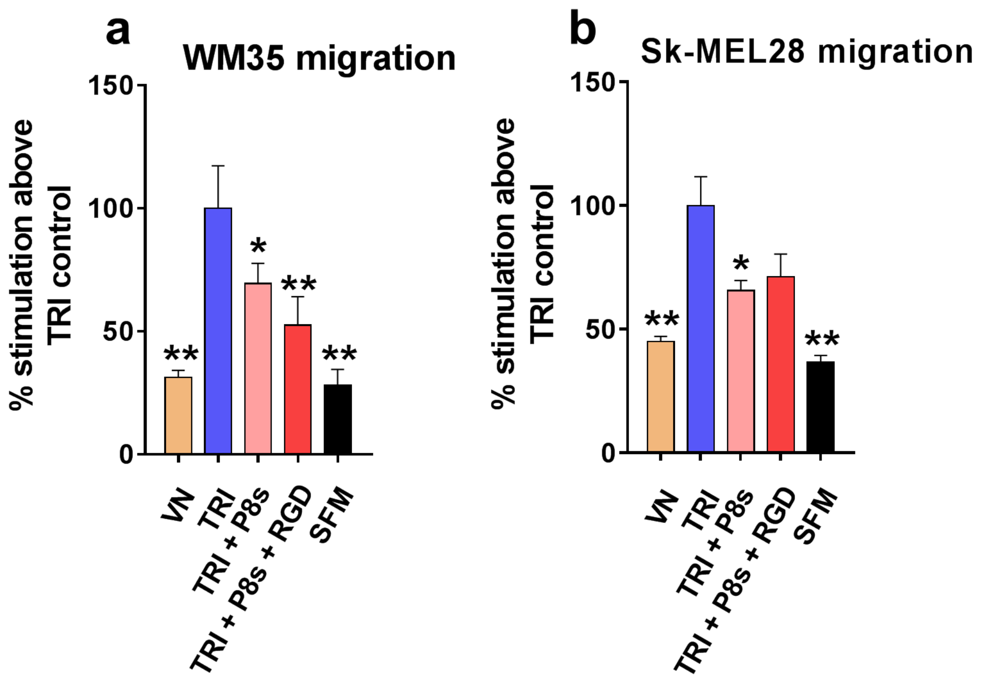

Supplementary Figure S7: Effect of the peptides P8s and RGD on TRI-stimulated cell migration. The lower chambers and under surfaces of Transwell plates were coated with VN alone or TRI supplemented with P8, P8s or RGD. Serum-starved WM35 (**a**) and Sk-MEL28 (**b**) cells were allowed to migrate for 15 hours. Data is expressed as percentage of cells that migrated compared to TRI control. P8s and RGD used was 100 nM and 50 uM, respectively. n = 2 (tested in

three wells in each experiment). TRI = 1 µg/mL VN + 100 ng/mL IGF-I + 300 ng/mL IGFBP-3. Error bars indicate SEM.

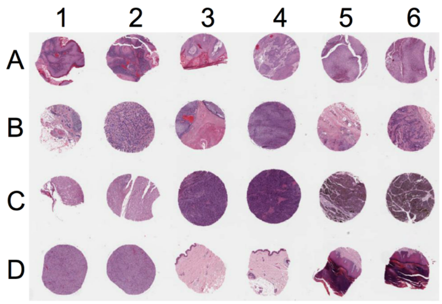

Position	Sex	Age	Organ	Pathology	Grade	Stage	TNM	Type
A1	F	77	Skin	Squamous cell carcinoma	I	I	T1N0M0	Malignant
A2	F	77	Skin	Squamous cell carcinoma	I	I	T1N0M0	Malignant
A3	M	72	Skin	Squamous cell carcinoma	I	I	T1N0M0	Malignant
A4	M	72	Skin	Squamous cell carcinoma	I	I	T1N0M0	Malignant
A5	M	67	Skin	Squamous cell carcinoma	II	II	T2N0M0	Malignant
A6	M	67	Skin	Squamous cell carcinoma	II	II	T2N0M0	Malignant
B1	M	44	Skin	Basal cell carcinoma	-	II	T2N0M0	Malignant
B2	M	44	Skin	Basal cell carcinoma	-	II	T2N0M0	Malignant
B3	M	55	Skin	Basal cell carcinoma	-	II	T2N0M0	Malignant
B4	M	55	Skin	Basal cell carcinoma	-	II	T2N0M0	Malignant
B5	F	47	Skin	Basal cell carcinoma	-	I	T1N0M0	Malignant
B6	F	47	Skin	Basal cell carcinoma	-	I	T1N0M0	Malignant
C3	M	49	Skin	Malignant melanoma	-	III	T3N0M0	Malignant
C4	M	49	Skin	Malignant melanoma	-	III	T3N0M0	Malignant
C5	M	36	Skin	Malignant melanoma	-	III	T3N0M0	Malignant
C6	M	36	Skin	Malignant melanoma	-	III	T3N0M0	Malignant
D3	F	50	Skin	Cancer adjacent skin tissue	-	-	-	Adjacent
D4	F	50	Skin	Cancer adjacent skin tissue	-	-	-	Adjacent
D5	M	73	Skin	Cancer adjacent skin tissue	-	-	-	Adjacent
D6	M	73	Skin	Cancer adjacent skin tissue	-	-	-	Adjacent

Supplementary Figure S8: Skin cancer tissue microarray and patient information

The human tissue microarrays (TMA) incorporating squamous cell carcinoma, basal cell carcinoma, malignant melanoma and cancer adjacent normal skin tissues were purchased from US Biomax Inc. (SK-242). The TMA slide was stained with H&E for morphological observations. Clinical information of the TMA cores were sourced from the manufacturer and tabulated. Grade 1-3 represents well-differentiated (low grade), moderately-differentiated (intermediated grade) or poorly differentiated (high grade) tumours, respectively, under microscopy. The TNM system was also used to describe the tumours based on the size and/or extent (reach) of the primary tumour (T), the amount of spread to nearby lymph nodes (N), and the presence of metastasis (M). T; T0: no primary tumour, T1-T4: size/extent of primary tumour. N; N0: no regional lymph node involvement, N1-N4: degree of regional lymph node

involvement. M; M0: no distant metastasis, M1: distant metastasis present. Clinical staging of stage I up to stage III is also included in the table.

Supplementary Materials and methods

3D GelMA assay: ImageJ quantification for spheroid size

Once the 3D cultures (Matrigel and GelMA) were fluorescently probed (with FDA) and images recorded, they were analyzed using ImageJ software (NIH). The scale was set in μm, after which the software converted image pixels into length (in μm). The number of spheroids and the size of each spheroid (in μm^2) within the image were calculated. Finally, average spheroid size for each treatment was calculated using the average size of all spheroids present in a single field of view and obtained from four fields of view.

Proximity ligation assay: ImageJ quantification for VN:IGFBP-3 transcript

In situ PLA of VN:IGFBP-3 complex were analysed by ImageJ. The threshold value was set to include PLA blobs and to omit background within the TMA cores. Circularity was set to cover blobs of 0.6-1.0 in circularity values. The number of PLA blobs was then quantified. Three such images from 3 different areas within a single TMA core were recorded for each cancer type and between non-tumor and tumor regions.
